# Defective Eyelids1Read before the June meeting of the Missouri Valley Veterinary Medical Association.

**Published:** 1900-07

**Authors:** R. C. Moore

**Affiliations:** Kansas City, Mo.


### DEFECTIVE EYELIDS.[1]

By R. C. Moore, D.V.S.,
KANSAS CITY, MO.

In the present discussion of this subject I desire to confine myself to three conditions that are most generally congenital, namely, ectropion, entropion, and blepharostenosis.

*Ectropion.* The name signifies an everted eyelid, either from an insufficiency of the skin of the lid, a destruction or shrinking of the same from injuries, an over-development of the mucous membrane, or insufficiency of the orbicularis palpebrarum muscle, either of which causes a displacement of the eyelid, turning its conjunctival surface outward, with, if it be the lower lid, a downward displacement of the ciliary margin, or an upward displacement of the same if it be an upper one. The eye, thus being deprived of its natural protection, is liable to chronic conjunctivitis, with all its accompanying evils. Ectropion is rare in equines and bovines, but common in the

[1] Read before the June meeting of the Missouri Valley Veterinary Medical Association.

canines; especially is this so in the St. Bernard and English bloodhound.

This condition is almost entirely confined to the lower lid, and its treatment depends largely on the cause. If it be from overdevelopment of the conjunctival membrane an elliptical piece of this should be removed in the following manner: The animal being placed under the influence of an anesthetic, the eyelid is everted and a portion of the membrane grasped with a pair of forceps and excised. The piece must be elliptical in shape and in size to correspond to the overdevelopment of the membrane. The outer incision must be parallel with the ciliary margin, and sufficiently far from it to allow room for suturing. The angles made by the counter incision, which is carried back of the forceps, should be near the inner and outer canthus. The incisions should be made straight through the membrane at a right angle to its surface so the edges will come together evenly. After the piece is dissected the edges of the membrane are to be brought together by sutures of a light, soft material placed so the knot will be as near as possible to the ciliary margin, where it will produce the least possible irritation to the eye, and left to heal by first intention, which should be assisted by mild non-irritating antiseptic dressings. The sutures should be removed about the fourth day.

If the lesion be simply tumefaction of the conjunctival membrane it may be penciled with nitrate of silver, by drawing the caustic from the inner to the outer canthus, just inside the ciliary membrane, wiping off the excess of caustic with cotton and protecting the parts by the application of a little bland oil.

The operation mentioned for overdeveloped membrane will be indicated if it be due to paralysis of the muscle. If due to insufficiency of the skin, it may be remedied by making a V-shaped incision with the base near the edge of the lid and the lid held in position by suturing the two lids together until the space between the edges of the incision is filled up by new tissue.

*Entropion.* The condition is the opposite of ectropion. The lid is inverted so as to bring the eyelashes in contact with the sensitive structures of the eye, causing traumatic conjunctivitis, with suppuration, granulation, adhesion, and finally, loss of sight. This condition is very much more common than ectropion. It is found in all our domestic animals, but most frequently in the dog, in which animal there appears to be more or less of an excess of skin. I am of the opinion that it is much more prevalent than most practitioners are wont to believe. The irritation and consequent pain cause the sufferer to keep the eye closed, which increases the pressure on and injury to the eye. The inversion may be the entire length of the lid or only a small part of it may be inverted. It may be turned in so that not only the lashes but the hair covering the skin of the lid is in contact with the eye; but in many cases only a limited number of the lashes touch the sensitive structure—called by some "trichiasis," which is merely a modified degree of entropion, and, owing to the very slight anatomical defect, much more difficult to diagnose.

One would naturally think that diagnosis would be easy, as the abnormality is in plain view, but such is not always the case. The constant pain causes the animal to keep the eye more or less closed, which hides the lesion. On careful observation, when the animal is not expecting you to disturb it, the opening to the eye will appear to be too small, then by comparison the ciliary margin will be seen to be displaced inward to a greater or less degree.

The condition is, in all probability, always congenital, although it may become more aggravated from the constant contraction of the orbicularis palpebrarum muscle, and is due to overdevelopment of the skin of the eyelid or, rather, too much skin for the mucous membrane, allowing the latter to draw the margin of the lid inward. May be either or both lids, but most commonly the lower lid.

Treatment consists in the removal of an elliptical piece of the lid, the same as of the membrane in ectropion. It is well to take up a piece of the skin with the forceps to determine just how much it is necessary to remove to bring the margin to its normal position, then remove the piece included in the forceps by making an incision through the skin from the inner canthus parallel to and just far enough from the margin to allow room for sutures, carrying it to the outer canthus or further if the skin be turned in at the canthus, then commencing at the same point near the inner canthus a counter incision is carried on the opposite side of the forceps to join the first at the outer canthus and the circumscribed piece removed, the edges brought together by closely placed interrupted sutures and treated antiseptically as an open wound, or the entire part closed by antiseptic dressings. If the latter is desired and both eyes are involved only one should be operated on at one time, leaving the other for use by the animal. The stitches may be removed from the fourth to the seventh day. Complete anæsthesia and cleanliness are essential to a successful operation.

*Blepharostenosis.* This is a congenital malformation where the opening to the eye is wanting or diminished in size or the margins of the lids unite from granulation of their surfaces as a result of disease or injury, causing complete ankylosis of the lids. Partial stenosis at the outer canthus is often seen in connection with entropion.

The treatment is similar, whether stenosis is partial, complete, or from adhesion by granulation. If complete the skin and membrane must be divided between the lashes from one canthus to the other and the mucous membrane sutured to the skin with close, fine interrupted sutures, with a view of getting union of the membrane and skin with the least possible granulation. As the latter is liable to reunite the margins of the lids, as a further precaution a suture may be placed through the skin near the center of one or both lids and the skin of the adjacent parts above or below, or both, to draw the upper lid up and the lower one down, so as to prevent contact of these margins until cicatrization is complete.

I know of no operation that gives better results than those just described, and if carefully and skilfully done the results are uniformly good.

